# HiACC: Hinglish adult & children code-switched corpus

**DOI:** 10.1016/j.dib.2025.111886

**Published:** 2025-07-17

**Authors:** Shruti Singh, Muskaan Singh, Virender Kadyan

**Affiliations:** a SoCS, University of Petroleum and Energy Studies, Dehradun, Uttarakhand, India; b SCIES, Ulster University, Northland Road, Londonderry, UK

**Keywords:** Automatic speech recognition, Code-switching, Children speech, Adult speech, Hinglish corpus

## Abstract

Code-switching is the frequent alternation between two or more languages within a single utterance and is a widespread phenomenon among bilingual and multilingual speakers. In India, more than 250 million people are estimated to engage in code-switched communication, especially blending English with Hindi (Hinglish), making it one of the largest bilingual populations globally, making challenging for developing accurate and robust Automatic Speech Recognition (ASR) systems. Existing ASR models, typically trained on monolingual corpus, struggle with code-switched input due to a lack of large, balanced, and representative datasets—particularly for diverse age groups. Recent evaluations have shown that ASR models experience a relative increase in Word Error Rate (WER) of 30–50 % when exposed to code-switched speech compared to monolingual input. To address this resource gap, we introduce a benchmark Hinglish speech corpus, *HiACC,* to improve ASR performance in resource-constrained settings. While several monolingual Hindi and English corpus exist, publicly available code-switched datasets remain scarce, and none till date include children's speech. Our corpus fills this gap by providing the first code-switched Hinglish speech dataset with recordings from both adults and children. It comprises 3,318 audio segments from adult participants and 1,858 segments from children, covering 5.24 hours of read and spontaneous speech. The transcriptions include detailed annotations and code-switching tags to assist in linguistic and computational analysis. The corpus is publicly available at [https://zenodo.org/records/15551669], offering segmented audio and aligned transcripts for open research. We also present baseline ASR experiments, which show that standard models trained on monolingual data underperform by approximately 42 % WER on our test set, highlighting the complexity of the task. To our knowledge, this is the first publicly available resource on code-switched Hinglish speech encompassing both adult and child speakers, designed to catalyse progress in this challenging yet important area of speech recognition.

Specifications Table

**Table:** 

Subject	Computer Science, Signal Processing, Deep Learning, Speech Processing
Specific subject area	Automatic Speech Recognition (ASR), Speech Processing, ASR for Low-Resource Languages, Bilingual ASR, Code-Switching, Code-Mixing, Code-switching, Children’s ASR
Type of data	Raw digital audio files (WAV format) and corresponding text transcriptions (txt format), sampled at 16 kHz
Data collection	The Hinglish code-switched speech corpus was collected through recordings of: (1) spontaneous responses to daily-life questionnaires, (2) story reading, and (3) responses to image-based questions in Hindi and English. For adults, all questions were presented in English, and participants responded freely in a mix of Hindi and English (Hinglish). Sessions were conducted in a single sitting without formal breaks, allowing participants to answer at their own pace. **Inclusion criteria:** Healthy participants; children aged 6–14 years and adults aged 18–40 years, with basic proficiency in spoken Hindi and English. **Exclusion criteria:** Participants with health conditions or speech impediments. **Tools used:** Samsung smartphone with a Play Store voice recorder application for audio capture, Praat software for audio processing, Transformer-based model for transcription, Windows and Linux operating systems.
Data source location	Adult speech data: University of Petroleum and Energy Studies, Dehradun, Uttarakhand, India Children’s speech data: Government Primary School, Upper Kandoli, Dehradun, Uttarakhand, India
Data accessibility	Repository name: Zenodo Data identification number: 10.5281/zenodo.15551669 Direct URL to data: https://zenodo.org/records/15551669 Instructions for accessing these data: Open access for academic/research use under a CC BY-NC 4.0 license. Users must cite the dataset when used in publications.
Related research article	Ganji S, Dhawan K, Sinha R. IITG-HingCoS corpus: A Hinglish code-switching database for automatic speech recognition. Speech communication. 2019 Jul 1;110:76–89.

## 1 Value of the Data

• To the best of our knowledge, the HiACC corpus is the first to offer both adult and children Hinglish speech data, addressing a significant gap in multilingual ASR research. It includes 5.24 hours of annotated read and spontaneous speech, allowing researchers to explore age-specific speech patterns and improve ASR systems for both adult and children speakers in bilingual settings.
• With precise annotations marking code-switching points and token-level language labels, the dataset allows researchers to study the structural and sociolinguistic aspects of Hinglish speech. It supports analysis of intra and inter-sentential code-switching, shedding light on bilingual language dynamics in real-world contexts.
• The corpus provides valuable training and testing material for building robust ASR systems capable of handling spontaneous code-switched speech, which is a known challenge for existing monolingual-trained models. Researchers can benchmark ASR performance and test advanced techniques such as transfer learning and cross-lingual adaptation.
• HiACC includes baseline ASR experiments using Whisper and Wav2Vec2 models, providing immediate reference points for researchers aiming to improve ASR accuracy on code-switched Hinglish. This facilitates quick comparative studies and accelerates progress in multilingual speech technology.
• The dataset features audio recorded in clean (adult) and realistic (children) environments, with some natural background noise in the latter. This variability helps researchers to develop and test ASR systems that are resilient to different acoustic environments, reflecting real-world usage scenarios.

## 2 Background

**Fig. 1:**
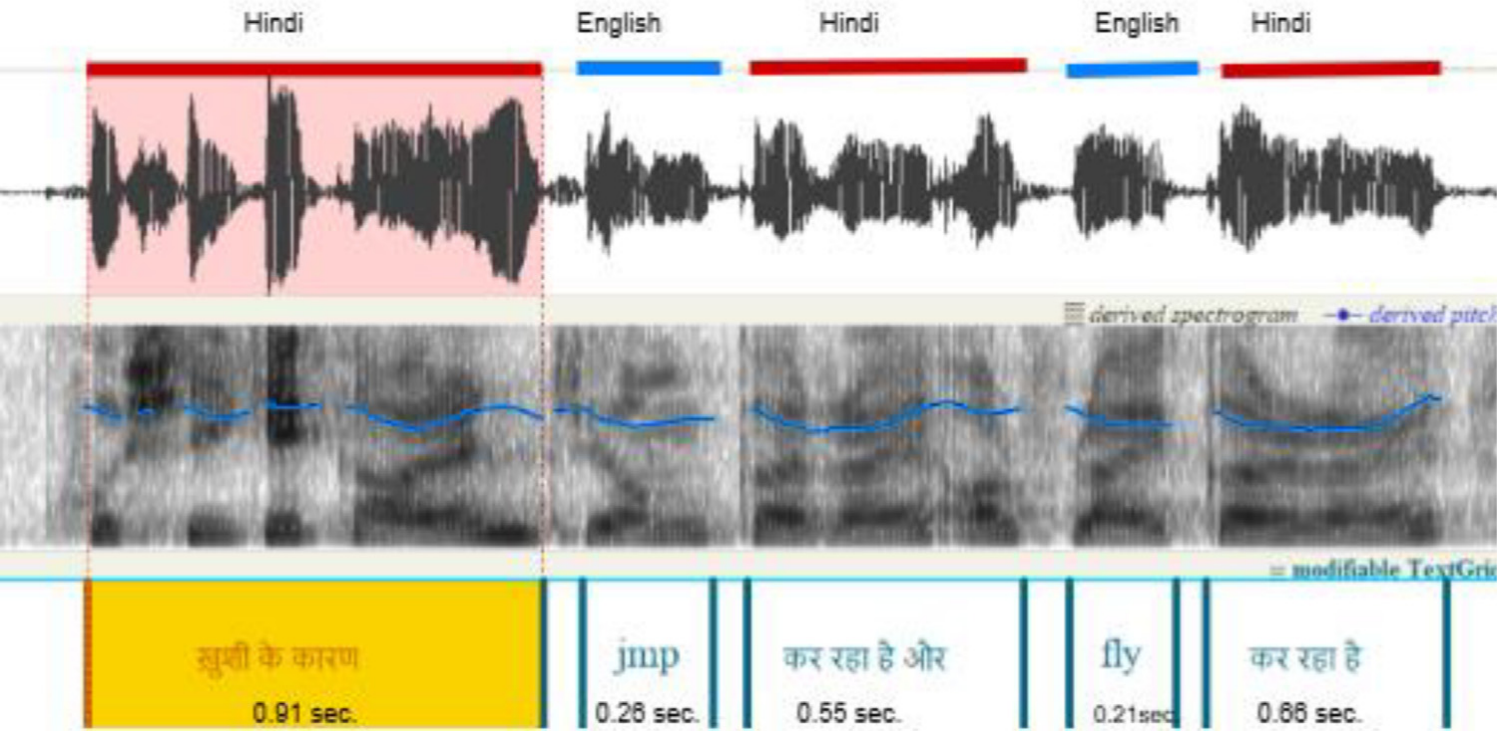
A sample of Hindi-English code-switching utterance from our dataset, with intra-sentential switching, namely Hindi-English-Hindi-English-Hindi, in 3.02 seconds.

*Code-switching*, the practice of alternating between two or more languages within a conversation, sentence, or phrase, is a widespread phenomenon in multilingual communities. It enhances communication by enabling speakers to express ideas more effectively and navigate between linguistic systems with ease [1]. Code-switching occurs in two main forms: *inter-sentential switching*, where the language alternates between sentences, and *intra-sentential switching*, where languages appear within a single sentence or phrase [2] as shown in Fig. 1. In India, *Hinglish* (a blend of Hindi and English) is a prominent example of code-switching, especially prevalent in urban and semi-urban areas. While Hindi is spoken as the first language by over *43%* of India's population, English is spoken as a second or third language by approximately *5.03%* of the population, with *2.64%* reporting English proficiency among Hindi speakers [3]. The rise of bilingual education, media exposure, and digital content has significantly increased English proficiency, particularly among younger populations, making *Hinglish* a frequent medium of communication. Globally, code-switching phenomena also occur in societies like Hong Kong (Cantonese-English), Malaysia (Malay-English), and the United States (Spanish-English). These multilingual environments present challenges for *Automatic Speech Recognition (ASR)* systems, which are typically trained on monolingual datasets. Studies have shown that ASR systems experience a *30–50%* increase in *Word Error Rate (WER)* when transcribing code-switched speech compared to monolingual speech, due to irregular grammar, informal vocabulary, and non-standard pronunciations [4]. Even state-of-the-art ASR systems like *Whisper* (OpenAI) and *Google’s multilingual models* struggle to handle spontaneous code-switching.

**Fig. 2:**
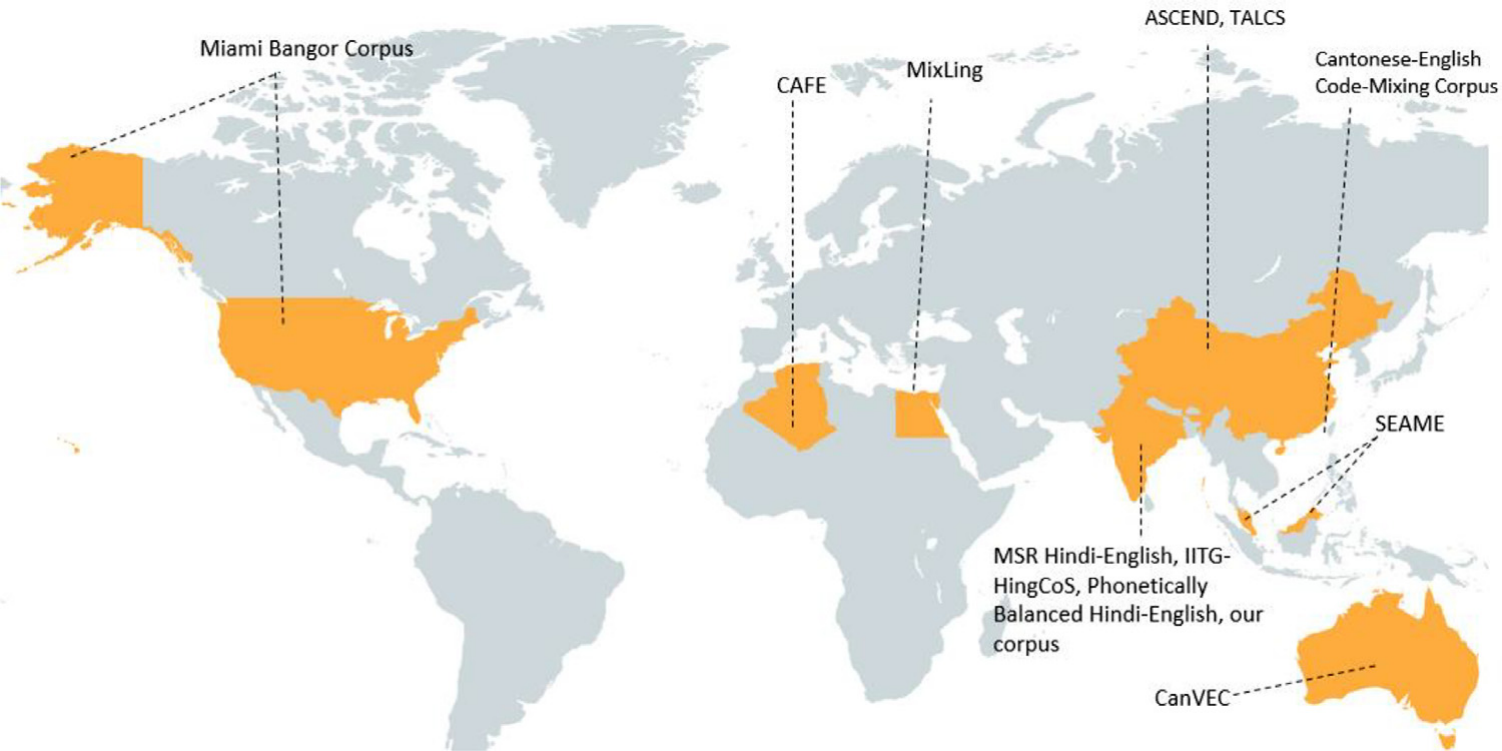
Geographic distribution of popular code-switched speech corpus across the world. The map highlights major datasets such as the Miami Bangor Corpus (USA), SEAME (Southeast Asia), CanVEC (Australia), CAFE (Europe), ASCEND and TALCS (China), MixLing (Germany), and the Cantonese-English Code-Mixing Corpus (Hong Kong). It also includes Hindi-English datasets such as MSR Hindi-English, IITG-HingCoS, the Phonetically Balanced Hindi-English corpus, and our proposed Hinglish speech corpus, primarily developed in India.

To address these challenges, several corpora have been developed for code-switched speech, such as the SEAME corpus (Mandarin-English) [5], the Miami Bangor corpus (Spanish-English) [6], the CAFE corpus (Algerian Arabic-French-English) [7], the ASCEND corpus (Chinese-English) [8] and the TALCS corpus (Mandarin-English) [12]. For Hinglish (Hindi-English), the IITG-HingCoS corpus [13] and the Phonetically Balanced Hindi-English Code-Mixed Corpus [14], which are primarily focused on adult speech and often involve *scripted or phonetically balanced content*. Fig. 2 illustrates the geographical distribution of these corpus, while Table 1 provides a comparative overview, detailing the language pairs, data types, speaker age groups, recording durations, and their public availability. To the best of our knowledge, no publicly available Hinglish corpus includes *children’s speech*, despite findings that ASR models trained on adult data tend to *underperform* when applied to children. This performance gap is primarily due to age-related differences in speech patterns, articulation, and fluency, leading to *error rates of 20–35% higher* for children’s speech [15].

**Table 1:** Overview of code-switched speech corpus categorized by language pair, data type, age group, duration, and publicly available status.

Corpus	Language Pair	Data Type	Age Group	Hours of Speech	Publicly Available
SEAME [5]	Mandarin-English	Spontaneous Speech	Adults	∼63 h	Yes
Miami Bangor Corpus [6]	Spanish-English	Conversational Speech	Adults	∼56 h	Yes
CAFE [7]	Algerian Arabic-French-English	Conversational Speech	Adults	∼15 h	Yes
ASCEND [8]	Chinese-English	Spontaneous Multi-turn Conversations	Adults	∼20 h	Yes
CanVEC [9]	Vietnamese-English	Natural Conversational Speech	Adults	∼10 h	Yes
Cantonese-English Code-Mixing Corpus [10]	Cantonese-English	Read + Conversational Speech	Adults	∼5 h	Yes
MixLing [11]	Arabic-English	Conversational + Dialectal Variation	Adults	∼12 h	Yes
TALCS [12]	Mandarin-English	Read + Spontaneous Speech	Adults	∼30 h	Yes
IITG-HingCoS [13]	Hindi-English (Hinglish)	Read + Spontaneous Speech	Adults	∼25 h	Yes
Phonetically Balanced Hindi-English [14]	Hindi-English (Hinglish)	Phonetically Balanced Read Speech	Adults	∼15 h	Yes
Our Corpus (this work)	Hindi-English (Hinglish)	Read + Spontaneous Speech	Adults + Children	5.24 h	Yes

In this paper, we address this challenge and present the *HiACC corpus*, a novel Hinglish code-switched speech dataset that includes both *adult* and *child speakers*. This dataset captures *naturalistic code-switching* through spontaneous responses to daily life questions, story reading, and image-based prompts. The corpus consists of *5.24 hours* of *segmented audio*, comprising *3318 utterances* from adults and *1858 utterances* from children, all manually transcribed with *code-switching annotations*. By including both *read and spontaneous speech* from both age groups, the HiACC corpus offers (1) *a comprehensive resource* of code-switched Hinglish speech that facilitates ASR research and benchmarking, incorporating both *adult and child speakers.* (2) *advance the development of ASR models* that are robust in *bilingual and multilingual contexts*, particularly in *low-resource environments*, by addressing the challenges of code-switched speech. (3) *support linguistic and sociolinguistic analysis* of *code-switching across age groups*, enhancing the understanding of bilingual speech patterns in *real-world communicative contexts*. (4) *contribute to the design of inclusive technologies*, particularly *ASR systems* that effectively handle *children’s speech* in bilingual settings, thereby improving the accessibility of speech recognition systems. We envision this corpus enabling advancements in multilingual ASR, particularly for applications in education technology, digital assistants, and accessibility tools.

## 3 Data Description

This section provides a detailed overview of the *Hinglish code-switched speech corpus,* which contains a collection of audio recordings with corresponding transcriptions. The corpus includes *audio recordings from both adult and child speakers*. Two types of speech are included: (a) *Spontaneous speech* consists of speakers responding to questions about their daily lives in Hinglish. The questions prompt speakers to describe images or share their thoughts on various topics, capturing natural, conversational code-switching between Hindi and English. (b) *Read speech* involves speakers reading a *predefined story*, which alternates between Hindi and English, showcasing code-switching in a structured context. The speakers in the corpus are *native Hindi speakers* from *northern India,* fluent in both *Hindi* and *English*. The *age range* for adult participants is between *18 and 40 years,* while child participants are aged between *6 and 14 years*. For the adult subjects, the recording sessions range from *6 to 23 minutes; for* children, sessions typically last from *5 to 11 minutes.* The statistics of the corpus used here are listed in Table 2

**Table 2:** The overall statistics of the code-switching Hinglish speech corpus are collected. Speaking rate is measured by the number of words per minute. Here, (M: Male, F: Female).

CS Speaker Type	Adult	Children
Number of Speakers	24 (M: 15, F: 9)	20 (M: 10, F: 10)
Number of Utterances	2668	1858
Number of Hours	3.22	2.04
Speaking Rate (words/min)	172.6	153.78
Age Group (years)	19 - 42	10–14
Native Language	Hindi	Hindi
Recording Environment	Clean	Clean

**Fig. 3:**
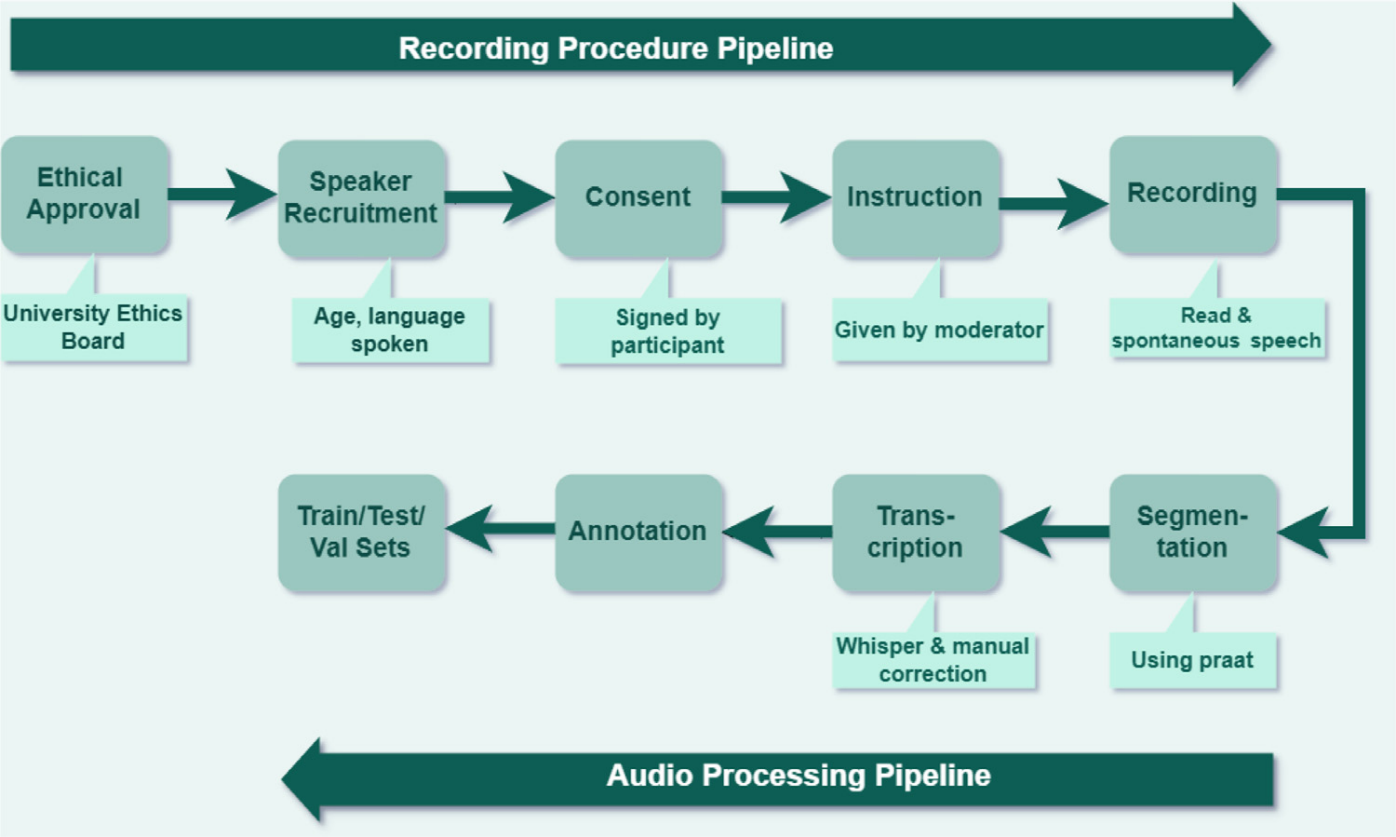
Pipeline for collection and processing of code-switched Hinglish speech corpus.

Fig. 3 illustrates the *two-stage pipeline* developed for creating the Hinglish speech corpus, which consists of the *Recording Procedure Pipeline* and the *Audio Processing Pipeline.* (a) *Ethical Approval* from the *University Ethics Board* is required to ensure compliance with all research standards. Following approval, participants are recruited based on *specific criteria*, including age and language proficiency in Hindi and English. (b) After obtaining *informed consent* from all participants, they are guided through the recording process by a moderator. All the participants engage in *two types of speech tasks:* spontaneous conversation and read speech. The recordings are conducted in a *clean environment* to ensure minimal noise interference. (c) After recording, the audio is processed using *Praat,* a tool to segment the speech into manageable utterances. To ensure accuracy, these segments are transcribed using the *Whisper ASR model and manual corrections*. (d) The annotated data includes *linguistic labels* (e.g., code-switch markers) and *metadata* (e.g., speaker ID). This allows the corpus to provide comprehensive insights into both the *linguistic structure* of Hinglish speech and the *speaker characteristics.* (e) Finally, the corpus is divided into *training, validation, and test sets*, ensuring the proper application of the dataset in training *ASR models.* This structured approach guarantees the *ethical sourcing, diversity, and completeness* of the Hinglish speech data, supporting robust analysis and modelling of *code-switched speech*.

### 3.1 Data collection

Our HiACC code-switching corpus consists of both *inter-sentential* and *intra-sentential* language switching, reflecting real-world code-switching between *Hindi* and *English*. The data collection process was initiated after receiving *ethical approval* from the *University’s Internal Ethical Committee*, ensuring adherence to participant privacy, informed consent, and ethical guidelines.

*Participant Recruitment and Recording Process* participants were selected based on predefined inclusion and exclusion criteria. The *inclusion criteria* required participants to be *healthy* individuals, specifically: *(a) Children aged 6 to 14 years, (b) Adults aged 18 to 40 years,* with basic proficiency in *speaking* and *reading* both *Hindi* and *English*. To ensure the clarity and consistency of the recorded speech, individuals with any *speech disorders* were excluded from the study. Prior to participation, each participant provided *informed consent* by signing a consent form. The form clearly stated that their participation was voluntary, they could withdraw at any time, and their data would be *anonymous.* The recordings took place in *two phases:* (a) *Read Speech*: Participants read text prompts aloud, where the text was designed to include both Hindi and English code-switching. (b) *Spontaneous Speech:* Participants responded freely to questions based on their *daily lives* and *personal experiences*, providing natural examples of *code-switching* in conversation.

*Session Setup and Environment* For both types of speech, participants interacted with the recording interface independently. The setup consisted of a *laptop* placed on a table with a *13.4-inch screen,* positioned approximately *20 to 30* cm from the participant’s eyes. A *PowerPoint presentation* displayed the prompts, and participants were instructed to proceed to the next question only after completing the previous one. During the session, participants were not interrupted, ensuring a *natural flow* of speech. The speech was recorded using a *Samsung Galaxy M34 5* G mobile phone with the *Voice Recorder* application. Audio was captured in *WAV format* at a *16* kHz *sampling rate* and *mono channel* settings.

*Recording Environments* The adult and children recording environments were different. *For the Adult Recordings, we* conducted them in a *university computer lab,* which provided a controlled environment for high-quality audio capture. While *children’s recordings* were recorded in a *classroom* environment, isolated from other activities. The classroom was located near roads and playgrounds, and some *background noise* from *vehicles* and *outdoor activities* was captured during the recordings. The isolated environment helped ensure that the recordings accurately represented *spontaneous* and *read speech* while accounting for some environmental noise, which is typical in real-world scenarios. Despite minor background noise, the overall data quality was maintained, and the corpus reflects the *natural, real-world use of Hinglish* in adults and children.

### 3.2 Pre-processing

The speech recordings collected were further pre-processed to prepare the data for ASR with *audio segmentation*, which was carried out using the *Praat tool* [16] to divide the continuous speech recordings into audio units based on utterance boundaries. We segmented based on both **syntactic** and **prosodic features** of the utterances.

**For the scripted speech (read speech),** segmentation was performed at line endings where syntactic boundaries occur. We divide the long audio recordings into smaller chunks, each corresponding to a single, complete utterance that may involve Hindi-English language switching. For instance, the utterance, “so चिंटू और राजू ने अपने bags में कुछ snacks और पानी pack किया और गुफा की तरफ चल दिए,” was treated as a single segment, as it forms a coherent syntactic unit. However, in cases where the speaker did not pause at line breaks or full stops and continued reading into the next line, segmentation was performed at the natural pauses in speech instead. Secondly, we dealt with spontaneous **speech,** which poses an additional challenge due to the absence of clear sentence boundaries using **prosodic cues**, *notable pauses* in the speaker’s flow, which help maintain the *natural rhythm* of speech, even in the absence of formal sentence boundaries. We split the longer utterances that could not be easily segmented syntactically, we split at points where the speaker made *prominent pauses*, ensuring coherent and natural audio segments.

**Fig. 4:**
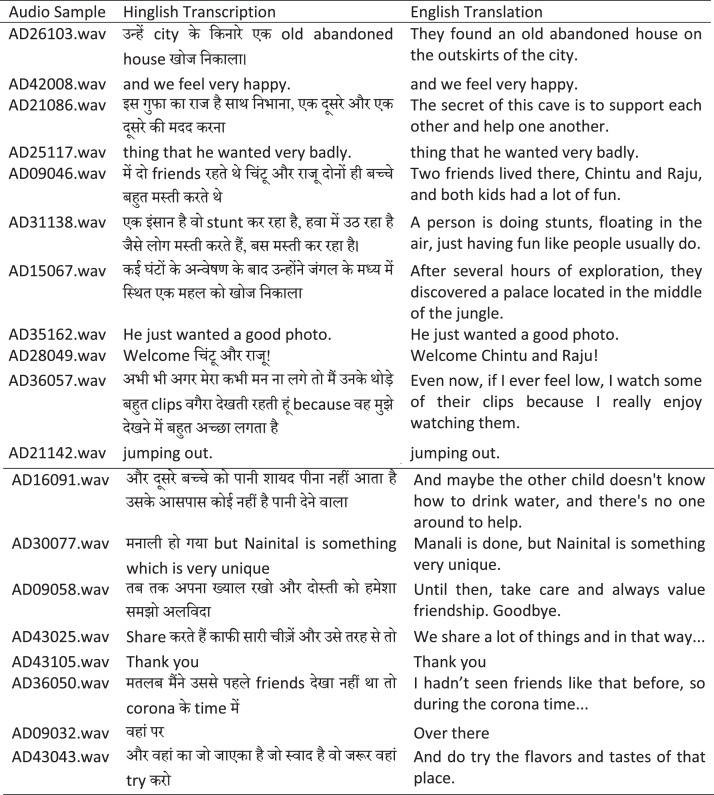

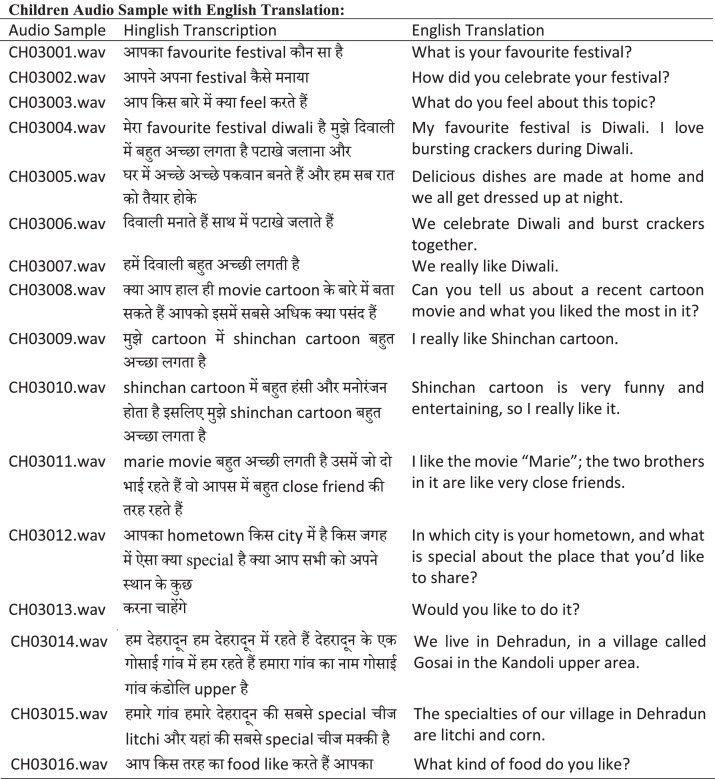
Example from HiACC speech corpus illustrating audio segments recorded from both adult and child participants.

To ensure consistency and facilitate downstream ASR processing, speech segments were kept short and uniform in length, with most segments being well-suited for effective model training and evaluation. We illustrate an instance of the segmentation Fig. 4, where the transcriptions are broken down into individual segments, each representing a distinct utterance or unit of speech.

### 3.3 Transcription

Further these segmented audio files were transcribed using both Devanagari script for Hindi words and Latin script for English words using the Whisper model. Following the initial transcription, we performed manual corrections to ensure the transcriptions were accurate and aligned to the original speech. The transcription process strictly adheres to *an accurate representation* of what was spoken, without altering the speech to meet grammatical conventions. For instance, if a participant uttered "recommended," the transcription reflects this exact utterance rather than the grammatically correct form "recommend." The intention was to maintain *fidelity to the spoken word* rather than modifying it for grammatical correctness. Additionally, *repeated words* and *speech errors* (both grammatical and semantic) were transcribed as heard. For example, *if a speaker repeats a word, it was transcribed twice:* "*I see for the peace, I don’t.*" In cases where a speaker uses *non-standard or repeated phrases*, these were also transcribed verbatim.

For example: “*और एक लड़का किसी डब्बे डुब्बे के सहारे पर अपना सहारा लेकर खड़ा हैं* (Here, “*डब्बे डुब्बे*” is transcribed exactly as heard, instead of the standard *डब्बे*”.)

These initial transcriptions were further corrected from a *linguistic perspective,* specifically when the audio quality was poor or the speaker’s accent was heavy, the ASR output was deemed inaccurate, and transcriptions were instead manually generated from scratch. However, these instances were rare in our corpus, and most of the corrections involved refining the ASR output rather than starting a new one.

### 3.4 Transcript structure

We followed a transcription structure with each transcription stored in a *text file*, with each line representing a *single speech sample*. The first column of the file lists the *path* or *name* of the corresponding *WAV file*, and the second column contains the transcribed speech text, separated by a comma. An example is illustrated in Fig. 4.

### 3.5 Metadata annotation and corpus partitioning

After the transcription and corrections were completed, we annotated the data with relevant metadata-**Speaker age** (adult or child), **Speech type** (read or spontaneous) and **Linguistic attributes** (code-switching markers, speech features). Finally, we divided this annotated corpus into ***training, validation, and test sets****,* facilitating the development and evaluation of ASR models. We followed a structured pipeline to ensure our corpus is of the *highest standardised* and *ethically sourced*, with rich linguistic data suitable for *cross-lingual speech processing research.* Additionally, we have *converted all numerical digits into words* to maintain consistency and avoid ambiguity in transcription.

### 3.6 Final layout of the hiacc corpus

**Fig. 5:**
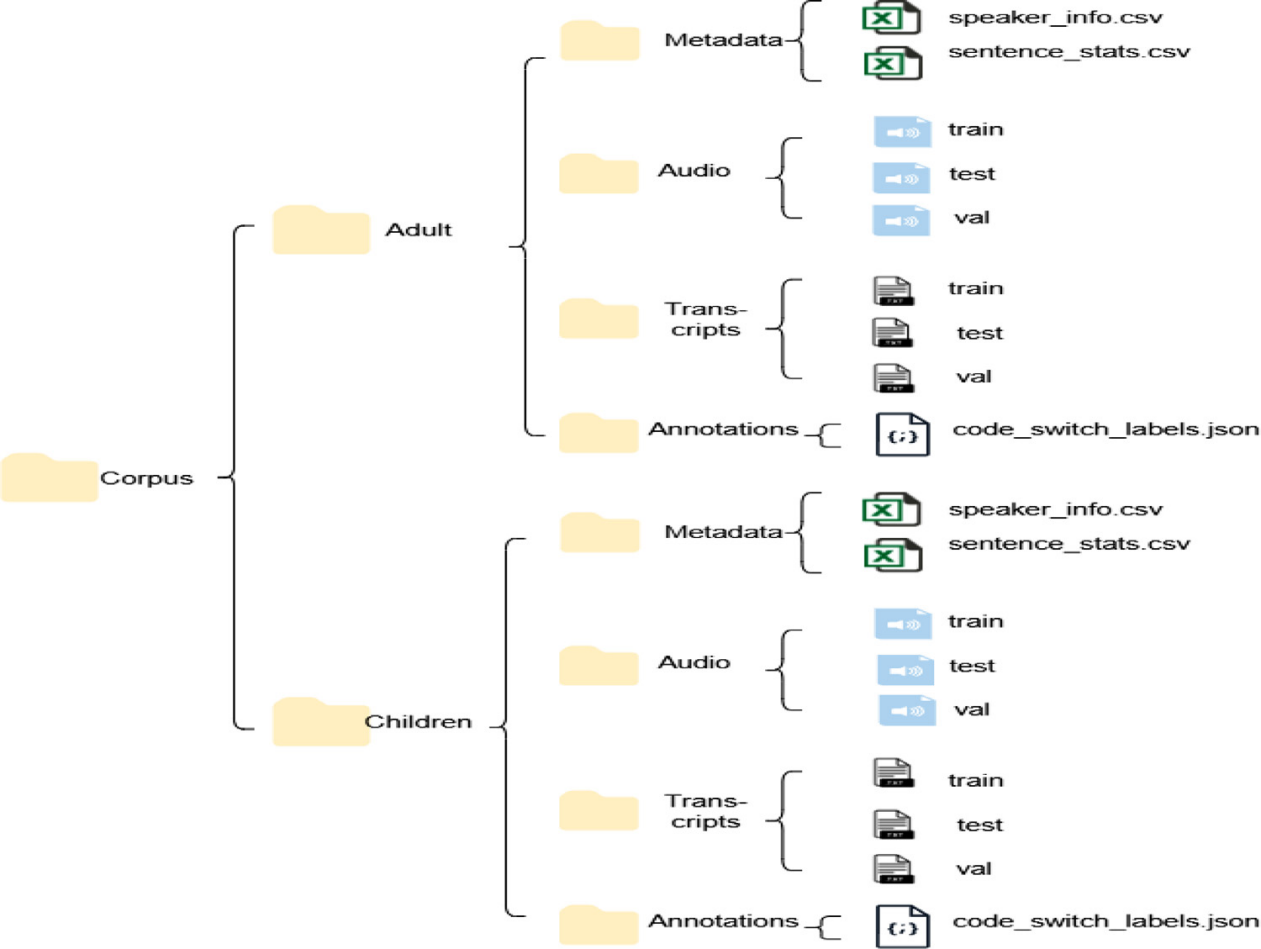
Corpus directory structure.

The dataset is organised into a structured directory to facilitate scalability and ease of use for various speech processing tasks. The directory structure, depicted in Fig. 5, is designed to ensure efficient access to data while supporting diverse applications in speech recognition and linguistic research.

The root directory, named *HiACC*, is divided into two main subdirectories: (1) **Adult** contains data related to adult speech. (2) **Children** contains data related to children’s speech. Each of these subdirectories follows a consistent organisational structure, with the following four main subdirectories:

• **metadata** folder includes two key CSV files: (a) *speaker_info.csv* contains demographic and linguistic information for each speaker, such as age, gender, and language proficiency. (b) *sentence_stats.csv provides utterance-level statistics*, such as sentence length and frequency of code-switching, which are useful for detailed analysis of speech patterns.
• **audio** directory is further subdivided into *train, test, and val* subfolders, each containing corresponding *.wav files*. These subsets were created using a *speaker-independent split*, ensuring that the data can be used to train and evaluate models that generalise well across unseen speakers.
• The **transcripts** folder mirrors the structure of the audio directory and contains the corresponding *transcriptions* for each audio file. These transcriptions are essential for training ASR models and conducting linguistic analysis.
• The **annotations** folder contains *code_switched_labels.json*, which provides detailed *token-level language labels* for each utterance. This file marks whether a token belongs to Hindi or English, with *Token-level language identification and/or Fine-grained analysis* of code-switching behaviour in speech.

This structured directory layout ensures that the data is well-organised and easily accessible for various applications, including *speech recognition, code-switching analysis,* and *linguistic research.*

## 4 Experimental Design, Materials and Methods

In **HiACC**, we explore both the linguistic and acoustic characteristics of code-switching behaviour and speech complexity across adult and child speakers. The adult corpus consists of 24 speakers (15 males, 9 females) and 3318 utterances totalling 3.22 hours of speech, recorded in a clean, controlled environment (e.g., a quiet lab setting). In contrast, the children’s corpus was recorded in a real-world environment, school classrooms, where occasional background noise (e.g., vehicle sounds and playground activities) was present. This corpus includes 20 speakers (10 males, 10 females), with 1858 utterances, amounting to 2.04 hours of speech. All participants were native Hindi speakers, with adults aged 19–42 years and children aged 10–14 years. The recordings were captured using a mono-channel setup and stored in WAV format at a sampling rate of 16 kHz and a bit depth of 16 bits, using Pulse Code Modulation (PCM) encoding. Both recordings followed consistent parameters across age groups, with both read and spontaneous utterances, ensuring a balanced representation of scripted and natural speech. This setup enables the reliable comparison of acoustic characteristics and supports robust ASR system development for code-switched Hinglish speech.

### 4.1 Acoustic analysis

The acoustic specifications for both adult and children speech are provided in Table 3. Both corpora were recorded in *mono-channel format* with *WAV file* format at *16* kHz *sampling rate* and *16-bit depth,* using *Pulse Code Modulation (PCM)* encoding. The *adult speech* was captured in a *clean, controlled environment,* such as a quiet lab, while *children's speech* was recorded in a *real-world environment,* school classroom, where *background noise* (e.g., vehicle sounds and playground activities) was occasionally present. Despite the difference in environment, the consistency in *recording parameters* ensures comparability of *acoustic characteristics* and enables effective training and evaluation of *ASR systems* designed to handle *code-switched Hinglish speech.*

**Table 3:** Details of acoustic specification including speaker type, audio format, sampling settings, and utterance type.

Speaker Type	Channel	Audio Format	Sampling Rate	Bit Depth	Format	Environment type	Format Settings	Utterance Type
Adult	mono	wav	16 kHz	16 bits	PCM	Clean	Little/Signed	Read, spontaneous
Child	mono	wav	16 kHz	16 bits	PCM	Clean	Little / Signed	Read, spontaneous

### 4.2 Linguistic analysis

The linguistic characteristics of the Hinglish speech corpus reveal distinct patterns of code-switching and speech complexity between the adult and child speakers. Table 4 shows the linguistic statistics of the recorded corpus. The adult corpus consists of 3318 utterances, which include 16,172 Hindi words and 16,307 English words, with 1322 unique Hindi words and 1865 unique English words. In contrast, the children corpus contains 1858 utterances, which include 9976 Hindi words and 2599 English words, with 1075 unique Hindi words and 513 unique English words. When it comes to code-switching behaviour, adults produced 1207 sentences with code-switching, which resulted in a total of 3539 code-switching instances. Meanwhile, children exhibited 1122 sentences containing code-switching, resulting in 3361 code-switching instances. Both groups showed a balanced number of Hindi→English and English→Hindi switch pairs. Specifically, adults produced 1665 Hindi→English switch pairs and 1877 English→Hindi switch pairs, while children produced 1637 Hindi→English switch pairs and 1724 English→Hindi switch pairs. In terms of speech rate, adults spoke at a rate of 172.6 words per minute, while children spoke at a slower rate of 153.78 words per minute. Unique switching patterns were also observed: adults had 1110 unique Hindi→English switch pairs and 1254 unique English→Hindi switch pairs, while children recorded 679 unique Hindi→English switch pairs and 693 unique English→Hindi switch pairs. The percentage of intra-sentential code-switched utterances was significantly higher among children (60.71%) compared to adults (36.65%), indicating that children exhibited more frequent language alternation within sentences. The Code-Mixing Index (CMI), which measures the degree of language mixing within an utterance, was calculated to be 25.91 % for adults and 21.11 % for children, indicating a higher degree of language mixing in adults' speech. Regarding fluency, adults demonstrated a faster speech rate of 0.39 seconds per word, while children had a slower rate of 0.45 seconds per word. The average sentence length was fairly similar for both groups, with 9.82 words per sentence for adults and 9.59 words per sentence for children. The corpus also captured common Hindi-English and English-Hindi switching pairs, such as “उनकी friendship” and “describe कर”, which are useful for developing more context-aware ASR models. These frequent switching patterns provide insights into natural code-mixing behaviour in Hinglish and are crucial for improving the performance of ASR systems designed to handle code-switched speech. Table 5 is showing the frequent switches occurred in the corpus.

**Table 4:** Linguistic statistics of the recorded corpus, including sentence and word counts, switching patterns, code-mixing metrics, and utterance-level properties across participants.

Speaker type		Adult	Child
# of sentences		3318	1858
# of total words	Hindi	16172 16307	9976 2599
	English		
# of unique words	Hindi	1322 1865	1075 513
	English		
# of sentences containing code-switching		1207	1122
# of code-switching instances		3539	3361
# of Hindi → English switch pairs		1665	1637
# of English → Hindi switch pairs		1877	1724
# of Hindi → English unique switch pairs		1110	679
# of English → Hindi unique switch pairs		1254	693
Average audio per speaker		138.25	92.9
% Code-switched utterances		36.65%	60.71%
Code Mixing Index (CMI)		25.91%	21.11%
Speech rate (word/*sec*)		0.39 s	0.45 s
Average sentence length (in words)		9.82	9.59
Intra-sentential switching		1095	1091
Inter-sentential switching		113	30

**Table 5:** Top 20 frequent Hindi–English and English–Hindi code-switching word pairs observed in the corpus.

Hindi-English Switching Pairs		English-Hindi Switching Pairs	
Adult	Child	Adult	Child
उनकी friendship कुछ snacks उन्हें city पानी pack ने guardian एक task एक old उनका next गए task का secret दो siblings में while दो friends एक dusty भाई known इस image बहन admired थे veer है and है i	इस image को describe क्या thoughts उनकी friendship का secret और Shyam मेरा favourite एक old उनका next कुछ snacks अपनी favourite से village की help और friendship मेरी favourite क्या special का food आपकी favourite और strong हैं next	city के friends रहते spot हो snacks और pack किया complete कर task के book मिली guardian के siblings रहते friendship बिल्कुल so चिंटू house खोज welcome चिंटू veer बड़े secret थोड़ा low उन्हें diya छोटी anxiously उन्होंने city दो	describe कर image को forest में city के cave का Shyam ने thoughts हैं city में favourite जगह house की snacks और friends रहते pack किया secret थोड़ा strong हो like करते village में spot हो secret को friendship और

### 4.3 Baselines

We experiment with baseline models for evaluating our code-switched speech corpus. The dataset was randomly divided into *70% for training, 20% for testing*, and *10% for validation*. We utilized state-of-the-art models, including *Whisper* and *Wav2Vec2*, to generate benchmark results for pre-trained models on the *code-switched Hinglish corpus*. All experiments were conducted on a *Tesla V100-PCIE-32GB* GPU. The performance of the Automatic Speech Recognition (ASR) systems was evaluated using the *Word Error Rate (WER)* metric, a standard evaluation metric that quantifies the number of errors (substitutions, deletions, and insertions) in the transcriptions, normalized by the total number of words in the reference transcription.

For the baseline, we fine-tuned a pre-trained **Whisper model** [17], which was trained on 680,000 hours of multilingual data using an encoder-decoder Transformer architecture. Specifically, we used the Whisper-medium checkpoint, which consists of 24 layers and 769 M parameters. During fine-tuning, we did not specify the languages of our corpus explicitly; instead, we allowed the model to identify the languages itself. Whisper is designed to handle multiple tasks, such as transcription, translation, and language identification, within a unified framework. For fine-tuning, we specified the task as “transcription”. The Whisper model performed well on the code-switched Hinglish corpus, achieving a WER of 16 % for adults and 18 % for children, as shown in Table 6. These results demonstrate Whisper’s ability to handle code-switched speech in Hinglish effectively, even without explicit language labels during training.

**Table 6:** Different Baseline Models on the Code-switched Hinglish Speech Corpus. Scores are presented in Word Error Rate (WER) after being categorized by the test set.

Speech Model	Code-switched Adult (WER %)	Code-switched Children (WER %)
Whisper medium	16	18
Wav2vec2-xls-r-300m	38	40
Mms-1b-all	31	36

The **Wav2Vec2 model** [18], released by Facebook AI in 2020, has shown superior performance on the LibriSpeech dataset. To expand its multilingual capabilities, a version called XLSR (Cross-Lingual Speech Representations) [19] was introduced, enabling the model to learn across multiple languages. XLSR was trained on nearly 500,000 hours of audio in 128 languages, with a model size scaling up to two billion parameters for enhanced cross-lingual performance. In addition, the Massive Multilingual Speech (MMS) [20] model, a recent release from Meta AI, covers over 1,000 languages and extends the capabilities of multilingual models, especially for low-resource and endangered languages. For our experiments, we fine-tuned both the Wav2Vec2-XLS-R-300 M and MMS-300 M versions of the Wav2Vec2 model on the adult and children corpus. Both models were fine-tuned with 300 M parameters. The XLS-R model achieved a WER of 38% for adults and 40 % for children, while the MMS adapter training achieved a WER of 31% for adults and 36% for children, as detailed in Table 6. These results indicate that both Wav2Vec2 models provide competitive performance on code-switched Hinglish speech, with the MMS model performing slightly better for both adult and children speech.

## Limitations

While the *HiACC corpus* provides a valuable resource for code-switched Hinglish speech, it has several *limitations* that should be considered when using it for research or model development.

(1) The overall size of the dataset, while substantial, remains relatively small, especially when compared to other large-scale multilingual corpus. The adult corpus consists of *3.22 hours* of speech, and the children’s corpus includes *2.04 hours*. Although these datasets are sufficient for preliminary experiments, they may not capture the full variability of code-switching patterns that occur in *larger, more diverse real-world settings.* A larger dataset could lead to better generalisation and improved performance in *ASR model training*. (2) The dataset exhibits a *gender imbalance* in terms of the number of male and female speakers. In the adult group, there are *15 males* and *9 females*, and in the children’s group, there are *10 males and 10 females.* While the children group is balanced in terms of gender, the *adult group* has a noticeable male skew. This imbalance could affect the *generalizability* of models, as gender-related speech patterns may vary, particularly in spontaneous speech where individual traits, including speech patterns, are more pronounced. (3) Several of the *children’s audio recordings* were captured in *real-world environment***s**, such as *school classrooms,* which introduced *background noise*, including sounds from vehicles and playground activities. While efforts were made to ensure high-quality recordings, this noise can impact *acoustic clarity* and may affect the performance of *ASR systems* when applied to real-world, noisy environments. Future efforts could involve better *noise suppression* or capturing data in more controlled environments to mitigate this issue. (4) The *age range of children participants* in this corpus is limited to 10-14 years, which restricts the dataset’s ability to represent younger children or those in other stages of *speech development*. Children’s speech evolves significantly as they grow, and the absence of a *more diverse age rang****e*** among children participants means that the dataset may not fully capture the variation in *language acquisition and speech complexity* across different developmental stages. Including a broader age range would help create a more comprehensive dataset for studying *age-related speech patterns* and would benefit the development of *age-adaptive ASR systems*. (5) The corpus predominantly focuses on *code-switched speech*, and while it does capture both Hindi and English, it lacks sufficient samples of *monolingual speech* in either language. This makes it less suitable for training ASR models that are intended to handle purely monolingual inputs, which are common in real-world applications where speech is often fully in one language. Including a larger portion of monolingual speech could help improve model performance across more varied language contexts. (6) Although the dataset includes both *spontaneous* and read speech, it may not fully capture the diversity of *code-switching behaviour* in different social, cultural, and linguistic contexts. Code-switching is highly context-dependent, influenced by factors such as *social setting, emotional state,* and *relationship* between speakers. This dataset may not represent all potential use cases of Hinglish, especially in highly informal or domain-specific contexts. Expanding the corpus to include more varied conversational settings would provide a richer understanding of *contextual code-switching*.

## Ethics Statement

Ethical approval for this study was granted by the Research Ethics Committee of the University of Petroleum and Energy Studies, Dehradun, India (REF-1002). All participants in the code-switched Hinglish corpus provided voluntary participation. No personal information, such as phone numbers or email addresses, was collected. Prior to participation, all participants were informed about the purpose of the data collection and gave their consent. They were provided with a clear explanation of the study and were required to sign a consent form confirming their understanding that their identities would remain anonymous. Participants were also informed that they could withdraw from the study at any time, and that their data would be treated confidentially and anonymously. The study adhered to ethical guidelines ensuring the protection of participants’ privacy and integrity. **Registration #**: EC/NEW/INST/2022/2820 and **Project #**: UPES/IEC/JUNE2024/1.

## CRediT authorship contribution statement

**Shruti Singh:** Conceptualization, Data curation, Writing – original draft. **Muskaan Singh:** Conceptualization, Supervision, Writing – review & editing, Investigation, Validation. **Virender Kadyan:** Supervision, Writing – review & editing, Investigation.

## Data Availability

ZenodoHiACC: Hinglish Adult & Children Code-switched Corpus (Original data)
